# Supplementary material for: Hot spot prediction in protein-protein interactions by an ensemble system
Source: BMC Syst Biol. 2018 Dec 31;12(Suppl 9):132. doi: 10.1186/s12918-018-0665-8 (PMC6311905; doi:10.1186/s12918-018-0665-8)
Supplement: Supplementary file 2 — All relevant MATLAB code. A simple Matlab implement of our predictor. (ZIP 19,742 kb) [file 12918_2018_665_MOESM2_ESM.zip › Additional file 2/Read ASA/ASA Property/SKEMPI/2O3BB.txt]

 NetSurfP - Protein Surface Accessibility and Secondary Structure Predictions

  

|  |  |  |
| --- | --- | --- |
|  |  | NetSurfP - Protein Surface Accessibility and Secondary Structure Predictions  Technical University of Denmark |

  


---

```
# For publication of results, please cite:
# A generic method for assignment of reliability scores applied to solvent accessibility predictions.
# Bent Petersen, Thomas Nordahl Petersen, Pernille Andersen, Morten Nielsen and Claus Lundegaard
# BMC Structural Biology 2009, 9:51 doi:10.1186/1472-6807-9-51
#
# Column 1: Class assignment - B for buried or E for Exposed - Threshold: 25% exposure, but not based on RSA
# Column 2: Amino acid
# Column 3: Sequence name
# Column 4: Amino acid number
# Column 5: Relative Surface Accessibility - RSA
# Column 6: Absolute Surface Accessibility
# Column 7: Z-fit score for RSA prediction
# Column 8: Probability for Alpha-Helix
# Column 9: Probability for Beta-strand
# Column 10: Probability for Coil
E S  2O3BB                  1    0.905 106.066  -0.194   0.003   0.003   0.994
E T  2O3BB                  2    0.642  89.059  -0.860   0.181   0.016   0.803
E K  2O3BB                  3    0.630 129.591  -0.133   0.502   0.002   0.495
E T  2O3BB                  4    0.540  74.926   0.316   0.600   0.003   0.397
E N  2O3BB                  5    0.312  45.721  -0.739   0.858   0.002   0.139
E S  2O3BB                  6    0.534  62.632  -0.301   0.923   0.002   0.076
E E  2O3BB                  7    0.553  96.522   1.247   0.923   0.002   0.076
B I  2O3BB                  8    0.082  15.096   0.868   0.970   0.001   0.030
B L  2O3BB                  9    0.123  22.521   0.447   0.970   0.001   0.030
E E  2O3BB                 10    0.574 100.278   1.486   0.970   0.001   0.030
E Q  2O3BB                 11    0.343  61.296   1.585   0.988   0.000   0.012
B L  2O3BB                 12    0.039   7.214   0.278   0.970   0.001   0.030
E K  2O3BB                 13    0.507 104.208   1.441   0.970   0.001   0.030
E Q  2O3BB                 14    0.552  98.569   1.120   0.923   0.002   0.076
B A  2O3BB                 15    0.101  11.108  -0.381   0.858   0.002   0.139
B S  2O3BB                 16    0.206  24.143  -0.919   0.600   0.003   0.397
E D  2O3BB                 17    0.570  82.094   0.524   0.246   0.004   0.750
B G  2O3BB                 18    0.271  21.304  -1.080   0.053   0.043   0.903
B L  2O3BB                 19    0.082  14.923   0.206   0.021   0.279   0.699
B L  2O3BB                 20    0.099  18.182  -0.433   0.021   0.451   0.528
B F  2O3BB                 21    0.122  24.425   0.563   0.022   0.359   0.619
B M  2O3BB                 22    0.166  33.317  -0.384   0.019   0.141   0.840
B S  2O3BB                 23    0.237  27.788  -1.187   0.018   0.088   0.893
E E  2O3BB                 24    0.412  71.976  -1.284   0.018   0.047   0.935
E S  2O3BB                 25    0.398  46.587  -1.153   0.053   0.043   0.903
E E  2O3BB                 26    0.476  83.122  -1.282   0.018   0.047   0.935
B Y  2O3BB                 27    0.210  44.962   0.608   0.018   0.047   0.935
E P  2O3BB                 28    0.318  45.067  -0.086   0.052   0.084   0.864
B F  2O3BB                 29    0.096  19.287   0.312   0.064   0.216   0.721
E E  2O3BB                 30    0.401  70.090   0.360   0.066   0.296   0.638
B V  2O3BB                 31    0.202  31.032  -0.553   0.135   0.317   0.548
B F  2O3BB                 32    0.134  26.994   0.202   0.148   0.418   0.435
E L  2O3BB                 33    0.375  68.608  -0.367   0.148   0.418   0.435
B W  2O3BB                 34    0.143  34.416  -0.246   0.064   0.216   0.721
E E  2O3BB                 35    0.584 102.025  -0.002   0.052   0.084   0.864
E G  2O3BB                 36    0.520  40.900  -2.456   0.018   0.019   0.964
E S  2O3BB                 37    0.602  70.590  -1.414   0.018   0.019   0.964
E A  2O3BB                 38    0.363  39.981  -1.389   0.018   0.019   0.964
E P  2O3BB                 39    0.460  65.331  -0.842   0.058   0.017   0.925
E P  2O3BB                 40    0.382  54.277  -0.888   0.257   0.016   0.727
B V  2O3BB                 41    0.155  23.747  -0.678   0.430   0.016   0.555
E T  2O3BB                 42    0.274  37.948   0.439   0.522   0.016   0.462
B H  2O3BB                 43    0.182  33.051  -1.343   0.923   0.002   0.076
E E  2O3BB                 44    0.375  65.478  -0.351   0.970   0.001   0.030
E I  2O3BB                 45    0.282  52.114   1.027   0.970   0.001   0.030
B V  2O3BB                 46    0.095  14.571   0.251   0.970   0.001   0.030
B L  2O3BB                 47    0.168  30.742   0.427   0.970   0.001   0.030
E Q  2O3BB                 48    0.520  92.943   0.403   0.923   0.002   0.076
E Q  2O3BB                 49    0.353  63.082   0.079   0.858   0.002   0.139
B T  2O3BB                 50    0.302  41.874  -0.141   0.502   0.002   0.495
E G  2O3BB                 51    0.625  49.211  -1.553   0.109   0.005   0.886
E H  2O3BB                 52    0.358  65.047   0.109   0.053   0.005   0.942
E G  2O3BB                 53    0.399  31.433  -0.921   0.018   0.019   0.964
E Q  2O3BB                 54    0.440  78.530  -0.522   0.058   0.017   0.925
E D  2O3BB                 55    0.674  97.138  -0.915   0.053   0.043   0.903
B A  2O3BB                 56    0.206  22.679   0.797   0.053   0.043   0.903
E P  2O3BB                 57    0.280  39.789  -0.071   0.118   0.150   0.732
B F  2O3BB                 58    0.137  27.496   0.381   0.148   0.418   0.435
E K  2O3BB                 59    0.552 113.588   0.730   0.079   0.592   0.329
E V  2O3BB                 60    0.356  54.671   0.592   0.169   0.612   0.219
B V  2O3BB                 61    0.201  30.848   1.214   0.169   0.612   0.219
E D  2O3BB                 62    0.459  66.142   0.807   0.135   0.317   0.548
B I  2O3BB                 63    0.091  16.853   0.880   0.605   0.105   0.290
E D  2O3BB                 64    0.486  70.061   0.252   0.660   0.049   0.291
E S  2O3BB                 65    0.377  44.220   0.268   0.660   0.049   0.291
B F  2O3BB                 66    0.098  19.649  -0.195   0.660   0.049   0.291
B F  2O3BB                 67    0.130  26.051   0.326   0.561   0.047   0.393
E S  2O3BB                 68    0.401  46.974   0.711   0.268   0.043   0.689
E R  2O3BB                 69    0.429  98.287   0.017   0.113   0.043   0.844
B A  2O3BB                 70    0.120  13.257   0.069   0.052   0.084   0.864
B T  2O3BB                 71    0.216  30.029  -0.983   0.056   0.142   0.802
E T  2O3BB                 72    0.321  44.578   0.120   0.018   0.088   0.893
E P  2O3BB                 73    0.465  65.998   0.252   0.052   0.084   0.864
E Q  2O3BB                 74    0.563 100.552  -0.312   0.053   0.043   0.903
E D  2O3BB                 75    0.711 102.441  -0.177   0.058   0.017   0.925
B W  2O3BB                 76    0.244  58.778  -1.453   0.058   0.017   0.925
E Y  2O3BB                 77    0.371  79.368  -0.174   0.115   0.016   0.868
E E  2O3BB                 78    0.543  94.792  -0.167   0.181   0.016   0.803
E D  2O3BB                 79    0.642  92.483  -0.395   0.522   0.016   0.462
E E  2O3BB                 80    0.573 100.121  -0.431   0.694   0.003   0.303
E E  2O3BB                 81    0.341  59.625   0.163   0.694   0.003   0.303
E N  2O3BB                 82    0.377  55.164   0.583   0.782   0.003   0.216
E A  2O3BB                 83    0.521  57.458   0.061   0.858   0.002   0.139
E V  2O3BB                 84    0.299  45.941   0.145   0.858   0.002   0.139
B V  2O3BB                 85    0.077  11.758  -1.008   0.858   0.002   0.139
E A  2O3BB                 86    0.457  50.372   0.887   0.858   0.002   0.139
E K  2O3BB                 87    0.380  78.084   0.952   0.858   0.002   0.139
B F  2O3BB                 88    0.041   8.249  -0.539   0.923   0.002   0.076
E Q  2O3BB                 89    0.349  62.385   1.317   0.970   0.001   0.030
E K  2O3BB                 90    0.443  91.105   1.573   0.970   0.001   0.030
B L  2O3BB                 91    0.028   5.127   0.704   0.970   0.001   0.030
B L  2O3BB                 92    0.115  21.002   0.738   0.988   0.000   0.012
E E  2O3BB                 93    0.522  91.106   1.410   0.988   0.000   0.012
B V  2O3BB                 94    0.135  20.719   1.325   0.988   0.000   0.012
B I  2O3BB                 95    0.028   5.254   0.871   0.970   0.001   0.030
E K  2O3BB                 96    0.402  82.650   1.747   0.970   0.001   0.030
E S  2O3BB                 97    0.612  71.762   1.214   0.858   0.002   0.139
B N  2O3BB                 98    0.238  34.843   0.333   0.694   0.003   0.303
B L  2O3BB                 99    0.057  10.473  -0.040   0.321   0.003   0.675
E K  2O3BB                100    0.594 122.206   0.713   0.115   0.016   0.868
E N  2O3BB                101    0.577  84.400   0.475   0.018   0.047   0.935
B P  2O3BB                102    0.123  17.411   0.845   0.020   0.205   0.775
E Q  2O3BB                103    0.359  64.153   0.791   0.023   0.655   0.322
B V  2O3BB                104    0.108  16.569   0.259   0.018   0.846   0.136
B Y  2O3BB                105    0.141  30.196   1.088   0.011   0.918   0.071
E R  2O3BB                106    0.315  72.066   0.530   0.018   0.846   0.136
B L  2O3BB                107    0.129  23.547   0.051   0.004   0.616   0.381
E G  2O3BB                108    0.322  25.365  -1.040   0.005   0.262   0.733
E E  2O3BB                109    0.544  95.054   0.096   0.005   0.262   0.733
B V  2O3BB                110    0.196  30.187  -0.074   0.004   0.616   0.381
E E  2O3BB                111    0.358  62.525   0.219   0.002   0.816   0.182
B L  2O3BB                112    0.045   8.294   0.762   0.001   0.900   0.099
B D  2O3BB                113    0.133  19.151   0.338   0.001   0.900   0.099
B V  2O3BB                114    0.027   4.088   0.818   0.001   0.959   0.040
B Y  2O3BB                115    0.066  13.997   0.587   0.001   0.959   0.040
B V  2O3BB                116    0.025   3.827   0.964   0.001   0.959   0.040
B I  2O3BB                117    0.033   6.068   0.323   0.001   0.900   0.099
B G  2O3BB                118    0.046   3.652  -0.823   0.003   0.718   0.279
E E  2O3BB                119    0.276  48.165   0.581   0.004   0.420   0.576
B T  2O3BB                120    0.140  19.376   0.488   0.004   0.138   0.858
E P  2O3BB                121    0.639  90.632  -0.358   0.018   0.088   0.893
E A  2O3BB                122    0.594  65.426  -1.406   0.018   0.047   0.935
E G  2O3BB                123    0.424  33.377  -1.350   0.018   0.019   0.964
E N  2O3BB                124    0.476  69.657  -0.270   0.018   0.088   0.893
B L  2O3BB                125    0.153  27.923   0.071   0.021   0.451   0.528
B A  2O3BB                126    0.157  17.257  -0.708   0.022   0.552   0.426
B G  2O3BB                127    0.185  14.567  -1.722   0.023   0.655   0.322
B I  2O3BB                128    0.130  24.087   0.351   0.021   0.756   0.223
E S  2O3BB                129    0.333  39.063   0.405   0.018   0.846   0.136
B T  2O3BB                130    0.252  34.925  -0.041   0.018   0.846   0.136
E K  2O3BB                131    0.353  72.674   0.848   0.011   0.918   0.071
B V  2O3BB                132    0.233  35.858  -0.109   0.011   0.918   0.071
B V  2O3BB                133    0.247  37.964  -0.182   0.018   0.846   0.136
E E  2O3BB                134    0.469  81.934  -0.278   0.004   0.616   0.381
E T  2O3BB                135    0.709  98.366  -0.553   0.003   0.003   0.994

Explain the output.  Go back.
```

---
